# LONELINESS AND HYPOCHONDRIASIS AMONG OLDER ADULTS: THE MEDIATING ROLE OF INTOLERANCE OF UNCERTAINTY AND ANXIETY

**DOI:** 10.1093/geroni/igz038.1954

**Published:** 2019-11-08

**Authors:** Jenna M Moore, William P Archuleta, Jessica H Helphrey, Leah N Smith, Jennifer Sawyer, David W Rose III, Christopher Reed, Michael D Barnett

**Affiliations:** 1 The University of Texas at Tyler, Tyler, Texas, United States; 2 University of North Texas, Denton, Texas, United States

## Abstract

Loneliness is prevalent among older adults and is associated with adverse outcomes for health and mortality. Additionally, researchers have suggested that loneliness may cause a person to direct attention inward and become preoccupied with bodily symptoms which may subsequently lead to health anxiety. However, little extant research has examined the association among older adults. In this study, we proposed a loneliness model of hypochondriasis in which loneliness contributes to hypochondriasis through intolerance of uncertainty and anxiety. Healthy, community-dwelling older adults (N = 280; 64.4% female; age range: 65-95; M = 76.08, SD = 7.59) completed an interview survey. Loneliness was associated with higher hypochondriasis and had an indirect effect on hypochondriasis through intolerance of uncertainty and anxiety. Lonelier older adults may have an activated threat system which prompts greater intolerance of uncertainty and anxiety and thereby results in greater hypochondriasis.

